# Supplementary material for: Intragastric Safflower Yellow Alleviates HFD Induced Metabolic Dysfunction-Associated Fatty Liver Disease in Mice through Regulating Gut Microbiota and Liver Endoplasmic Reticulum Stress
Source: Nutrients. 2023 Jun 29;15(13):2954. doi: 10.3390/nu15132954 (PMC10343935; doi:10.3390/nu15132954)
Supplement: Supplementary file 1 [file nutrients-15-02954-s001.zip › Fig.S1.pdf]

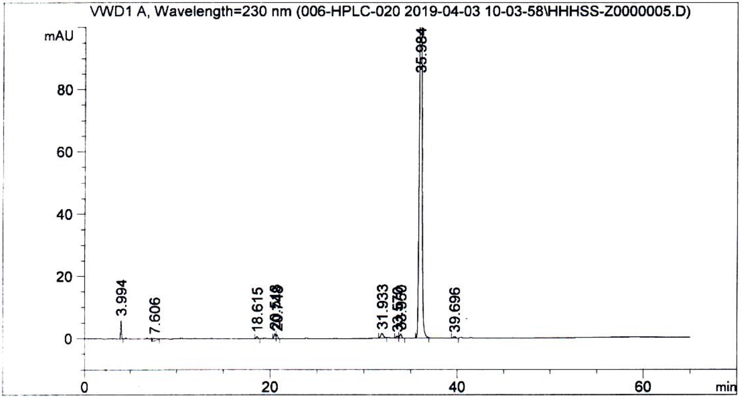

Area Percent Report

Sorted By : Signal
Multiplier : 1.0000
Dilution : 1.0000
Do not use Multiplier & Dilution Factor with ISTDs

Signal 1: VWD1 A, Wavelength=230 nm

Peak #	RetTime [min]	Type	Width [min]	Area [mAU*s]	Height [mAU]	Area %
1	3.994	BB	0.1031	39.64480	6.00055	1.3458
2	7.606	VB	0.3743	25.76117	8.65319e-1	0.8745
3	18.615	BB	0.1879	8.39716	6.95199e-1	0.2851
4	20.513	BV	0.1746	18.45795	1.59946	0.6266
5	20.748	VB	0.1677	13.08910	1.19625	0.4443
6	31.933	BB	0.2968	29.29592	1.48116	0.9945
7	33.570	BV	0.2201	8.88366	6.24065e-1	0.3016
8	33.950	VB	0.2261	18.34034	1.25803	0.6226
9	35.984	BB	0.2722	2776.21973	157.05194	94.2439
10	39.696	BB	0.2449	7.69147	4.96104e-1	0.2611

Totals : 2945.78130 171.26807
