# Supplementary material for: Intragastric Safflower Yellow Alleviates HFD Induced Metabolic Dysfunction-Associated Fatty Liver Disease in Mice through Regulating Gut Microbiota and Liver Endoplasmic Reticulum Stress
Source: Nutrients. 2023 Jun 29;15(13):2954. doi: 10.3390/nu15132954 (PMC10343935; doi:10.3390/nu15132954)
Supplement: Supplementary file 1 [file nutrients-15-02954-s001.zip › Fig.S2.pdf]

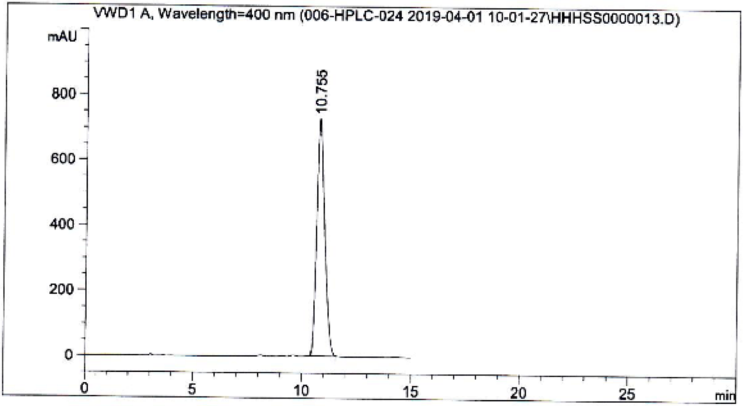

===

Area Percent Report

===

Sorted By : Signal
Multiplier : 1.0000
Dilution : 1.0000
Do not use Multiplier & Dilution Factor with ISTDs

Signal 1: VWD1 A, Wavelength=400 nm

Peak #	RetTime [min]	Type	Width [min]	Area [mAU*s]	Height [mAU]	Area %
1	10.755	BB	0.3720	1.84861e4	728.63824	100.0000

Totals : 1.84861e4 728.63824
