# Supplementary material for: Intragastric Safflower Yellow Alleviates HFD Induced Metabolic Dysfunction-Associated Fatty Liver Disease in Mice through Regulating Gut Microbiota and Liver Endoplasmic Reticulum Stress
Source: Nutrients. 2023 Jun 29;15(13):2954. doi: 10.3390/nu15132954 (PMC10343935; doi:10.3390/nu15132954)
Supplement: Supplementary file 1 [file nutrients-15-02954-s001.zip › Table S1.pdf]

Table S1: Primers for RT-qPCR

Gene	Forward primer	Reverse primer
m-*Fasn*	GGAGGTGGTGATAGCCGGTAT	TGGGTAATCCATAGAGCCCAG
m-*Acc*	GATGAACCATCTCCGTTGGC	GACCCAATTATGAATCGGGAGTG
m-*Srebp1*	TGACCCGGCTATTCCGTGA	CTGGGCTGAGCAATACAGTTC
m-*Ppia*	GCTGGACCAAACACAAACGG	TCCTGGACCCAAAACGCTC
m-*Scd1*	TTCTTGCGATACACTCTGGTGC	CGGGATTGAATGTTCTTGTCGT
m-*Hspa5*	ACTTGGGGACCACCTATTCCT	ATCGCCAATCAGACGCTCC
m-*Xbp1s*	ACGAGGTTCCAGAGGTGGAG	TGTCCAGAATGCCCAAAAGG
m-*Ppara*	AGAGCCCCATCTGTCCTCTC	ACTGGTAGTCTGCAAAACCAAA
m-*Cpt1a*	AACAACGGCAGAGCAGAG	CCACATAGAGGCAGAAGAGG
m-*Atf4*	ATGGCGCTCTTCACGAAATC	ACTGGTCGAAGGGGTCATCAA
m-*Atf6*	GACTCACCCATCCGAGTTGTG	CTCCCAGTCTTCATCTGGTCC
m-*Ddit3*	CTGGAAGCCTGGTATGAGGAT	CAGGGTCAAGAGTAGTGAAGGT
m-*Xbp1*	AGCAGCAAGTGGTGGATTTG	GAGTTTTCTCCCGTAAAAGCTGA
m-*Tnf*	TAGCCAGGAGGGAGAACAGA	TTTTCTGGAGGGAGATGTGG
m-*Il1b*	TTGAAGAAGAGCCCATCCTC	CAGCTCATATGGGTCCGAC
h-*SREBP1*	ACAGTGACTTCCCTGGCCTAT	GCATGGACGGGTACATCTTCAA
h-*ACC*	GATGAACCATCTCCGTTGGC	GACCCAATTATGAATCGGGAGTG
h-*SCD1*	TCTAGCTCCTATACCACCACCA	TCGTCTCCAACTTATCTCCTCC
h-*FASN*	GGAGGTGGTGATAGCCGGTAT	TGGGTAATCCATAGAGCCCAG
h-*TNF*	CCTCTCTCTAATCAGCCCTCTG	GAGGACCTGGGAGTAGATGAG
h-*IL1b*	ATGATGGCTTATTACAGTGGCAA	GTCGGAGATTCGTAGCTGGA
h-*PPARA*	CCAGTATTTAGGAAGCTGTCCTG	CGTTGTGTGACATCCCGACAG
h-*CPT1A*	TCCAGTTGGCTTATCGTGGTG	TCCAGAGTCCGATTGATTTTTGC
h-*ATF4*	TCGACTTGGATGCCCTGTTG	GGGAGATGGCCAATTGGGTT
h-*ATF6*	GTCAGCACCATCCCTGAGTC	CTCTTCAGTCTGGCAGGGTC
h-*DDIT3*	GGAAACAGAGTGGTCA TTCCC	CTGCTTGAGCCGTTCA TTCTC
h-*XBP1*	CCCTCCAGAACA TCTCCCCAT	ACA TGACTGGGTCCAAGTTGT

h-*GAPDH*	ACAACTTTGGTATCGTGGAAGG	GCCATCACGCCACAGTTTC

m: mouse, h: human
