# Supplementary figures and images for: Intragastric Safflower Yellow Alleviates HFD Induced Metabolic Dysfunction-Associated Fatty Liver Disease in Mice through Regulating Gut Microbiota and Liver Endoplasmic Reticulum Stress
Source: Nutrients. 2023 Jun 29;15(13):2954. doi: 10.3390/nu15132954 (PMC10343935; doi:10.3390/nu15132954)

Cell viability (%)

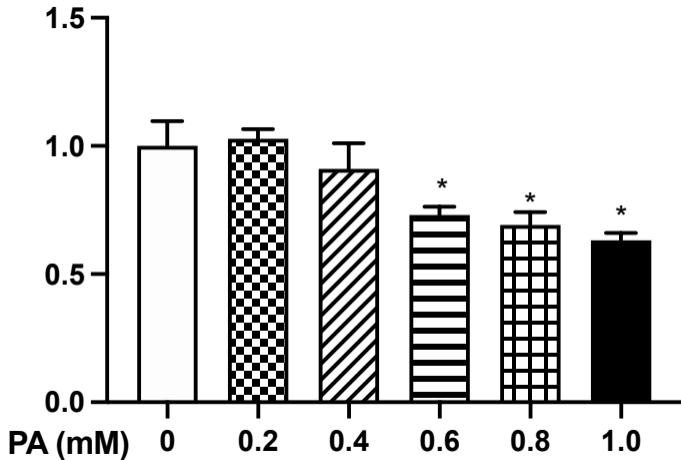

Supplement: Supplementary file 1 [file nutrients-15-02954-s001.zip › Fig.S3.pdf]

**A**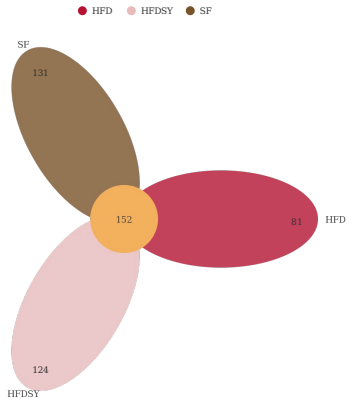**B**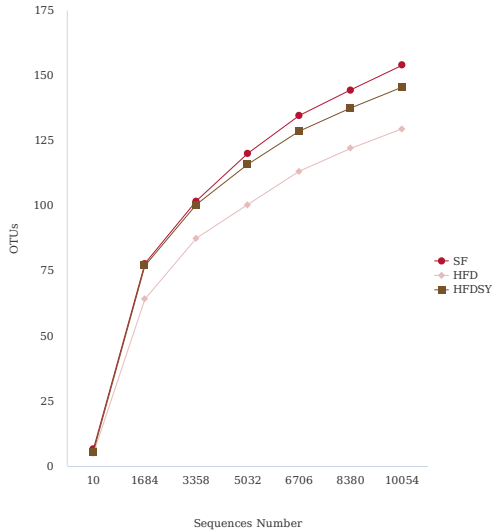

Supplement: Supplementary file 1 [file nutrients-15-02954-s001.zip › Fig.S4.pdf]
